# Spatial control of chemical processes on nanostructures through nano-localized water heating

**DOI:** 10.1038/ncomms10946

**Published:** 2016-03-10

**Authors:** Calum Jack, Affar S. Karimullah, Ryan Tullius, Larousse Khosravi Khorashad, Marion Rodier, Brian Fitzpatrick, Laurence D. Barron, Nikolaj Gadegaard, Adrian J. Lapthorn, Vincent M. Rotello, Graeme Cooke, Alexander O. Govorov, Malcolm Kadodwala

**Affiliations:** 1 School of Chemistry, University of Glasgow, Joseph Black Building, Glasgow G12 8QQ, UK; 2 School of Engineering, University of Glasgow, Rankine Building, Glasgow G12 8LT, UK; 3 Department of Physics and Astronomy, Ohio University, Athens, Ohio 45701, USA; 4 Department of Chemistry, University of Massachusetts, 710 North Pleasant Street, Amherst, Massachusetts 01003, USA

## Abstract

Optimal performance of nanophotonic devices, including sensors and solar cells, requires maximizing the interaction between light and matter. This efficiency is optimized when active moieties are localized in areas where electromagnetic (EM) fields are confined. Confinement of matter in these ‘hotspots' has previously been accomplished through inefficient ‘top-down' methods. Here we report a rapid ‘bottom-up' approach to functionalize selective regions of plasmonic nanostructures that uses nano-localized heating of the surrounding water induced by pulsed laser irradiation. This localized heating is exploited in a chemical protection/deprotection strategy to allow selective regions of a nanostructure to be chemically modified. As an exemplar, we use the strategy to enhance the biosensing capabilities of a chiral plasmonic substrate. This novel spatially selective functionalization strategy provides new opportunities for efficient high-throughput control of chemistry on the nanoscale over macroscopic areas for device fabrication.

## Abstract (web summary)

It is generally believed that rapid dissipation means that spatially precise heating is not feasible via thermoplasmonic means. Here, the authors induce highly localized heating around plasmonic nanostructures by pulsed laser irradiation, which effects chemical modification of surface bound molecules.

## Graphical abstract

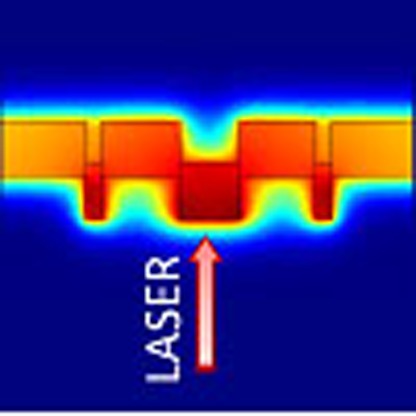

Incorporating molecular functionality into engineered nanomaterials is a requirement for the development of new sensing^1^,^2^,^3^, photovoltaic^4^ and optical technologies^5^,^6^,^7^,^8^. Maximizing coupling between nanostructures and molecular species requires spatial control of surface chemistry on the nanoscale. Currently, there are a variety of techniques that can be used to spatially control chemical functionality on nanostructured substrates such as dip-pen lithography^9^, inkjet printing^10^ and direct laser patterning^11^,^12^. However, all these approaches are in essence ‘top-down' methods for micrometre-scale substrate areas and each features limitations such as time-consuming processing and difficulties in achieving sub-50 nm resolutions. Furthermore, they require complex alignment procedures to be overlaid onto fabricated nanostructures.

In this study we show a high-throughput ‘bottom-up' approach that uses the nano-localized heating of a liquid (water) surrounding a nanostructure, to spatially direct a protection/deprotection strategy^13^. This novel strategy enables molecular materials to be placed in selective regions of a nanostructure. The protection step involves the chemical passivation of the gold structure with a self-assembled monolayer (SAM) of a long-chain polyethylene glycol (PEG) thiol. The spatially selective deprotection step then is the thermally driven structural transformation of the PEG SAM.

Structurally complex plasmonic nanostructures can produce a range of electromagnetic (EM) fields that not only have different spatial extents but also differing intrinsic properties. Consequently, for sensing purposes, it would be advantageous to selectively place material in locations occupied by EM fields with the desired properties. To this end, we have employed the nano-localized heating-driven protection/deprotection strategy to functionalize chiral plasmonic nanostructures, to enable selective positioning of proteins in a region with EM fields of enhanced chiral asymmetry. The placement of proteins in regions with fields of optimal properties provides enhanced biosensing performance, enabling attomole (∼6.0 × 10^5^ molecules) rather than femtomole (∼6.0 × 10^8^ molecules) detection levels.

## Results

### Modelling of nano-localized thermoplasmonic water heating

**Figure 1:**
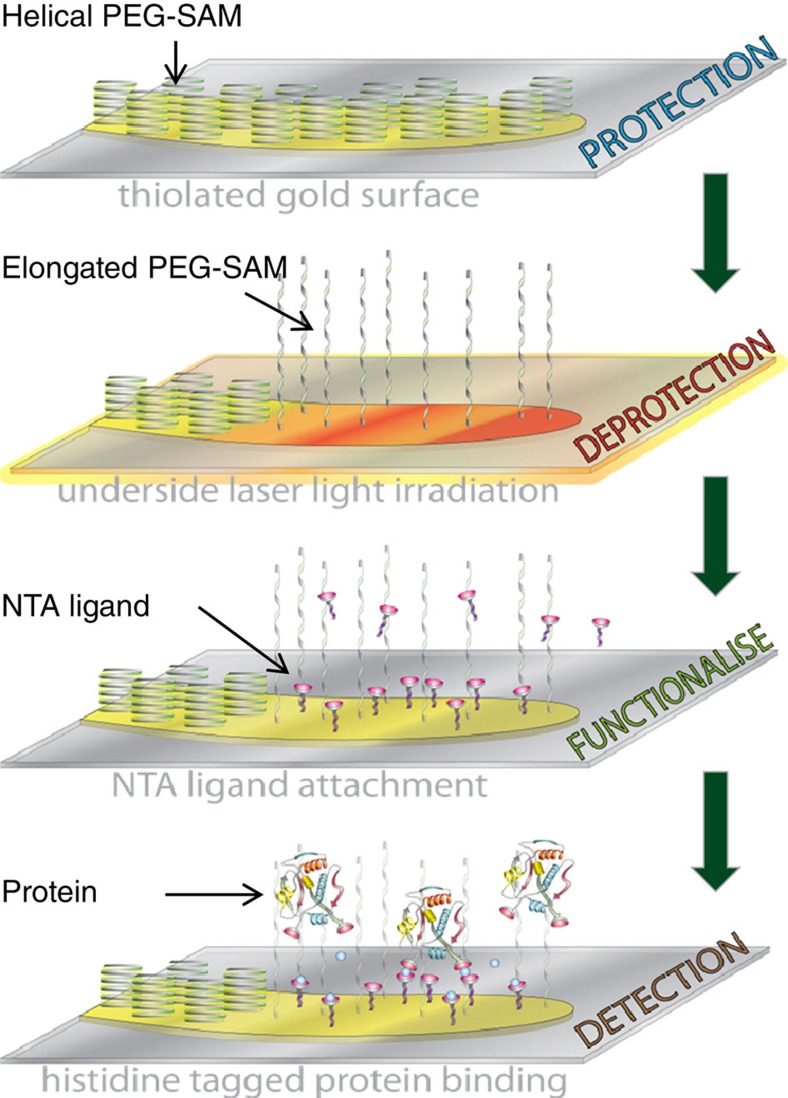
Pictorial representation of the steps in the protection/deprotection strategy. The nanostructure is first protected using a thermally responsive PEG-SAM (helical form). The second step is the deprotection where the nanostructure is irradiated by a nanosecond pulse laser and the subsequent localized water temperatures will cause a transition in the PEG-SAM to its elongated form. The elongated PEG-SAM will not inhibit the NTA ligands from attaching to the surface, thereby functionalizing the selective regions. Proteins can then be positioned in the particular regions for detection.

To date, it is believed that spatially localized chemistry on individual nanostructures cannot be achieved through thermoplasmonic phenomena, although thermal gradients can be generated in ensembles of nanoparticles separated by a poorly conducting medium^14^,^15^,^16^. This assumption is based on the fact that although electric fields, created by plasmon excitation, may be highly localized, efficient thermal diffusion in metal nanostructures results in rapid and uniform temperature increase throughout a particular structure in less than a nanosecond. This rapid heat dissipation leads to a uniform temperature at the nanostructure surface and hence spatial uniformity of surface chemical/physical processes. In this study we demonstrate a new phenomenon, which leads to spatially localized thermally driven chemistry, allowing nanoscale precision placement of biomaterial on a nanostructure surface (Fig. 1). Localized chemistry in this study is the result of significant temperature gradients in the surrounding water and not thermal gradients across the nanostructure surface itself.

**Figure 2:**
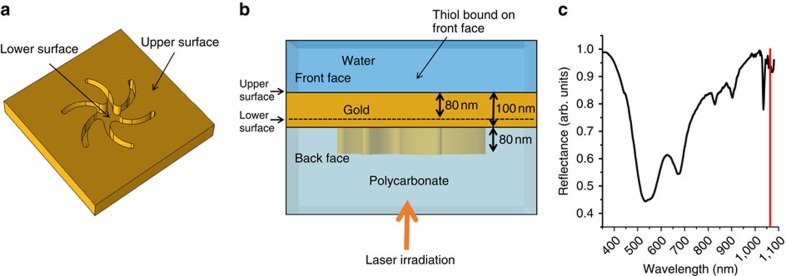
TPS experimental geometry and reflectivity. (**a**) Perspective view of the front face of the gold layer on a TPS. (**b**) Side view of the TPS, which is irradiated using a nanosecond laser through the polycarbonate. (**c**) Reflectivity of the TPS measured through the polycarbonate. The red line indicates the wavelength of the nanosecond laser.

We demonstrate this new ‘thermoplasmonic' effect using a chiral plasmonic nanomaterial formed using injection moulding, creating nanopatterned indentations in a polycarbonate slide (polycarbonate template) and coating the surface with a continuous Au film. The nanostructure in particular is a ‘shuriken' structure (Fig. 2a), which is either left or right handed and is referred to as a templated plasmonic substrate (TPS)^17^. These structures are 500 nm end to end and have a pitch of 700 nm from centre to centre. In contrast to traditional electron beam or photolithography, the TPS offer low-cost high-throughput fabrication, effectively a disposable consumable ideally suited for technological exploitation. The TPS are incorporated into a liquid cell with a total volume of *ca.* 100 μl. Interfaces that will subsequently be referred to as the front face, back face, lower and upper surfaces are defined in Fig. 2 (further details in Supplementary Fig. 1). In our approach, the back face of the TPS was irradiated using a 1,064-nm Nd:YAG laser with an 8-ns pulse.

**Figure 3:**
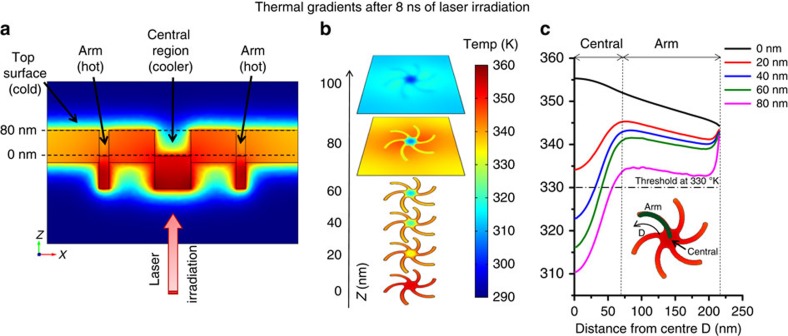
Surface plot of the thermal gradients at 8 ns (pulse end). (**a**) Side view showing gold and water regions and (**b**) stacked surface plots of water regions for increasing height *Z*. (**c**) Graph showing the temperature changes along a parametric curve passing along an arm to the centre (as shown by green line in inset) of the nanostructure for varying height *Z*. The horizontal line at 330 K represents the threshold for the deprotection step discussed.

On irradiation, the plasmonic structure absorbs the energy based on its resonance condition and the spatial regions of the nanostructure with high current density will then act as heat sources^18^,^19^. To understand this pivotal step, we have modelled the spatially resolved time dynamics for thermal behaviour of the nanostructure and the surrounding water for an 8-ns laser pulse at a fluence of 15 mJ cm^−2^. Unlike plasmonic heating with femtosecond pulses^20^, the gold film, owing to its high thermal conductivity, does not generate large thermal gradients (Fig. 3a) with nanosecond laser pulse durations^16^,^19^. Although the plasmonic fields maybe highly localized, the heat generated diffuses in the structure over such time scales (Supplementary Figs 3–6). With thermal diffusivity almost three orders of magnitude smaller than gold, the surrounding water does generate thermal gradients, developing three regions of varying thermal behaviour by the time the 8-ns pulse ends. The water at the top surface has a low average temperature but the two regions of water within the indentation itself have high temperatures over significant distances. The water in the central region of the indentation is cooler than that in the arms with increasing distance from the surface and the water temperature in the arms is relatively high and uniform with increasing height (Fig. 3a–c and Supplementary Fig. 3).

**Figure 4:**
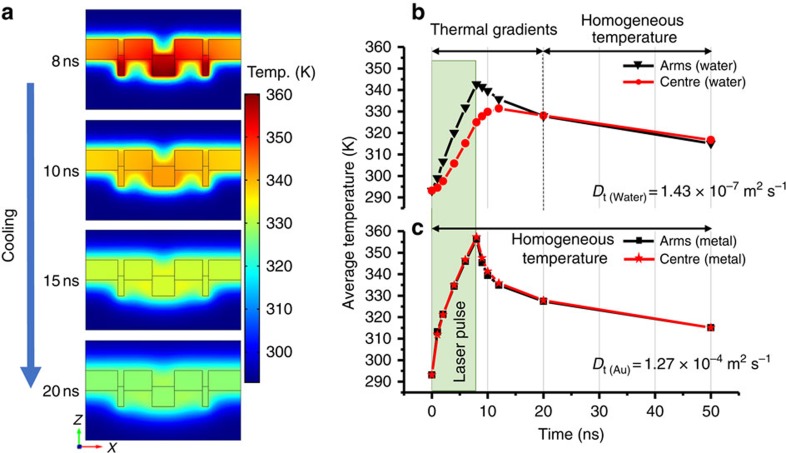
Thermal behaviour of water on irradiation. (**a**) Simulated spatially resolved temperature dynamics after pulse (8 ns) laser irradiation. (**b**) Average temperature of water (10 nm<*Z*<70 nm) and (**c**) metal (*Z*<0 nm) in the arm and centre regions. The values *D*_t_ are the thermal diffusivity of water and Au.

The time-dependant thermal behaviour shows that during the laser pulse, the water shows a steep increase in the average temperature for the two regions (Fig. 4) with the arm sections achieving higher temperatures than the central region, reaching a maximum at the end of the pulse (8 ns). This leads to thermal gradients in the water across the nanostructure that exist up to 12 ns after the pulse ends (that is, the arms are significantly hotter than the central region). After 12 ns, the water thermal gradients have completely disappeared and the water achieves thermal homogeneity with the metal as well. Equivalent thermal gradients do not exist in the metal (Fig. 4c). Thus, our simulations show thermal gradients in the water surrounding the nanostructure during the 8-ns laser pulse and a subsequent 4 ns afterwards. This phenomena derives from the disparity in the thermal diffusivities of water and Au. It is fundamentally different to the previously reported focusing of light into mesoscale volumes at water surfaces by Au nanoparticles, which creates a nanobubble of steam surrounding the resonantly heated nanoparticle surface^21^.

### Nano-localized thermoplasmonic chemical functionalization

The first step for our patterning process is the deposition of a protective SAM. To achieve spatially selective chemistry, we have used a thermally responsive SAM composed of a high-molecular-weight (6 kDa) PEG methyl ether thiol (PEG-thiol) polymer. The SAMs were formed by depositing PEG-thiol from solution onto the TPS; these substrates will subsequently be referred to as PEG-TPSs. The macromolecular structure of PEG displays a temperature dependency, which has been exploited previously to produce thermally responsive materials^22^,^23^. The macromolecular structure of PEG is governed by the conformation adopted by the –(CH_2_–CH_2_–O)– subunits. At low temperatures or in polar environments a gauche conformation is favoured, whereas at higher temperatures or in non-polar environments the *trans* form is observed^24^,^25^. Hence, PEG thiols adopt a compact ordered helical structure within SAM at low temperature and in aqueous solutions, which inhibit the adsorption of biomaterials. Driven by the conformational transition of the –(CH_2_–CH_2_–O)– subunits, PEG-SAM can undergo a transition from the helical state to an elongated form, which does not inhibit the adsorption of biomaterials^26^. To achieve this transition, Shima *et al*.^22^ have shown that the PEG-SAM must be exposed to temperatures over ∼330 K, along the entire length of its elongated structure, ∼35 nm (ref. 27).

**Figure 5:**
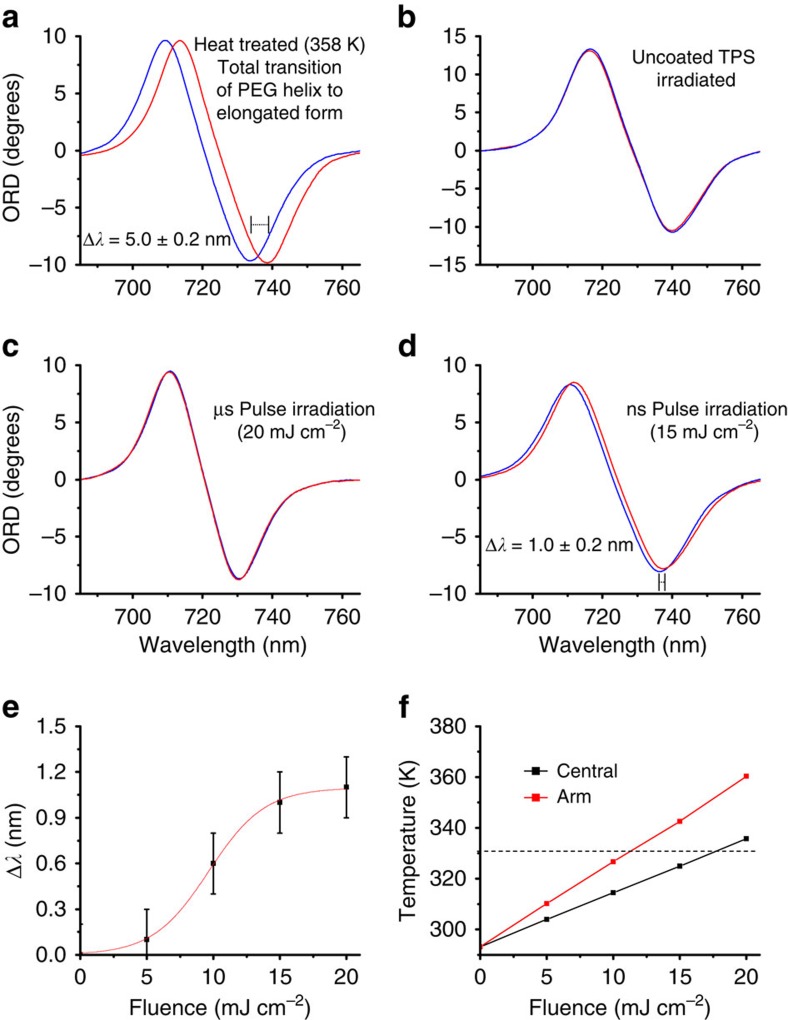
TPS nanostructure ORD measurements. (**a**) PEG-TPS heat treated at 358 K. (**b**) A plain TPS irradiated with 8 ns pulse laser; PEG-TPS irradiated using (**c**) 400 μs pulse laser and (**d**) 8 ns pulse laser. (**e**) Fluence dependence of the resonance shift and (**f**) simulation results for average temperature within the nano indentation for varying fluence.

The sensitivity of the wavelength of a plasmonic resonance to the refractive index of the near field enables change in the structure of the PEG-SAM to be monitored spectroscopically. We measured the shifts in the optical rotatory dispersion (ORD) of our left-handed plasmonic shurikens from the front face^17^. The helical and elongated *trans* form SAM have thickness of ∼13 and ∼35 nm, respectively^27^. Consequently, it would be expected that a transition from the helical to elongated *trans* form results in a significant increase in the thickness of the PEG-SAM layer and hence will cause a red shift in the plasmonic resonance. Indeed, when the PEG-TPSs are exposed either to water at 358 K (Fig. 5a) or a less polar solvent such as 1-butanol (see Supplementary Fig. 7), an irreversible red shift of +5.0±0.2 nm in the wavelength of the plasmonic resonance in ORD spectra is in fact observed. We attribute the irreversible nature of the transition to the kinetic effects of lateral interactions (such as intertwining) of the elongated *trans* PEG within the SAM that occur when temperatures are between 323 K (see Supplementary Fig. 7) and 358 K (Fig. 5a). When a PEG-TPS is heated to 358 K in air (for 48 h), no discernible shift within error (−0.2±0.2 nm) is observed (Supplementary Fig. 7). Hence, heating a PEG-TPS in air only, causes no change, indicating that the process is water mediated (Supplementary Fig. 7D)^27^.

The PEG-TPS were irradiated using a pulsed laser from the back and the energy dependence of the transition was evaluated. A plain TPS with no PEG-SAM shows no change (Fig. 5b) when irradiated using 8 ns pulse laser (15 mJ cm^−2^ fluence), indicating that the laser irradiation causes no deformation to the nanostructure. When a PEG-TPS is irradiated using 400 μs pulse laser, no observable changes occur (Fig. 5c) even at the highest fluence (20 mJ cm^−2^) used. When the PEG-TPS is irradiated using 8 ns pulse laser (15 mJ cm^−2^ fluence), a small shift of +1.0±0.2 nm is observed, indicating that a small fraction of the PEG-SAM has been changed (Fig. 5d). The level of red shift saturates within 60 s of laser exposure with further radiation causing no measureable change (Supplementary Fig. 7). When a substrate that has had its PEG transformed to the elongated state using 1-butanol is irradiated with nanosecond pulses, no spectral changes are observed (Supplementary Fig. 7). These data clearly demonstrate that nanosecond laser heating causes only a partial yet irreversible change to the PEG-SAM. Furthermore, the effects of laser irradiation show dependency on the fluence, displaying stepwise behaviour (Fig. 5e). Nanosecond and microsecond pulses can be considered to be quasi continuous, as they have significantly longer durations than the electron–phonon relaxation time^28^. Consequently, they only generate linear hot electron-driven phenomenon rather than the nonlinear phenomenon associated with femtosecond pulses, which arise through high electron temperatures. Although microsecond pulses would generate smaller field intensities, they would occur for longer time scales. Thus, the time integrated total flux of hot electrons will be identical for both nano and microsecond pulses^29^. Hence, the experimental observations are consistent with a thermal process rather than either a direct optical or hot-electron-mediated excitation, as both would have no dependence on pulse duration and a linear dependency on fluence^30^.

The small red shift (1.0±0.2 nm) induced by nanosecond pulse laser irradiation compared with the shift (5.0±0.2 nm) due to heating at 358 K indicates that only a small fraction (∼20%) of the total area in the near fields of the nanostructures of the PEG-SAM undergoes a transition to the elongated state. This limited transformation is consistent with our hypothesis that thermal gradients within the surrounding water can direct spatially selective chemistry. The irreversible transformation is a kinetically limited process that consequently requires the water temperature to be above the threshold for a sufficient time period, to enable the elongated PEG-thiol to intertwine with each other. Earlier studies of the dynamic relaxation for polymers in solution with similar molecular weight have shown this time period to be ∼5–10 ns (ref. 31). For shorter time periods, the transformation would be reversible. The results from our simulation show that at the top surface, the temperatures in the water are not high enough to achieve the conformational transition. Hence, the PEG-SAM here will remain in its helical conformation over the entire surface. At the central cooler region of the structure, the average water temperature barely crosses the threshold for <3 ns. The PEG-SAM here will not experience the required temperatures for a long-enough period and hence will also remain in a helical conformation. However, the arm region, is at a significantly higher average temperature (∼350 K) and above the threshold for ∼12 ns. Only in the arms is the average water temperature higher than the threshold for long enough to achieve the irreversible transformation.

The dependence of the resonance shift on the laser fluence is also in agreement with our simulation results on the dependence of temperatures on laser fluence (Fig. 5f). The average temperature in the arm only increases above the threshold for the PEG transition for a fluence of >12 mJ cm^−2^ and just about crosses that threshold in the centre at the highest fluence of 20 mJ cm^−2^, indicating that substantial transition of PEG-SAM will occur only in the arms.

**Figure 6:**
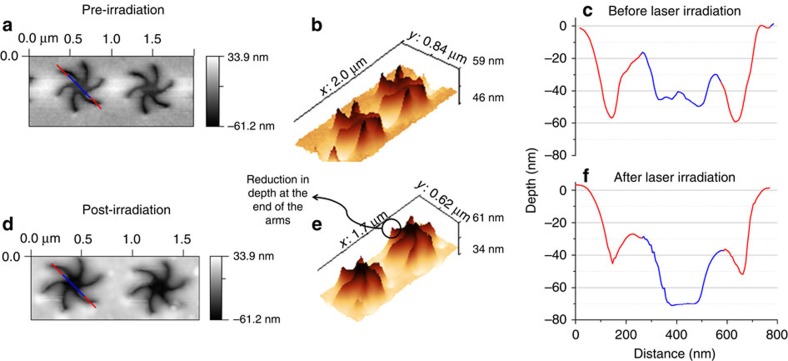
AFM of pre and post irradiation of the PEG-TPS. (**a**,**d**) The height plots, (**b**,**e**) the inverted 3D plots and (**c**,**f**) the height data of along the lines shown in **a** and **d**. The red and blue colours correspond to the distances marked in the SEM(**a**,**d**).

To confirm the spatial localization of the PEG-SAM transformation and hence the validity of using thermal gradients in water to illicit spatially selective chemistry, we have collected atomic force microscopy (AFM) images. The significant height difference between the helical and the elongated *trans* form (∼22 nm) of the PEG can readily be detected by AFM. In Fig. 6 are AFM images collected from PEG-TPS before and after irradiation with nanosecond pulses. The AFM images show that after laser irradiation there is an average reduction in the depth of the arms by 20–30 nm in the region at the end of the arms. This decrease in depth is an expected change in SAM thickness on going from helical to elongated *trans*-PEG. The spatial localization of the change in SAM structure is also supported by scanning electron microscopy, which show change in contrast at the end of the arms post irradiation (see Supplementary Fig. 8).

### Enhanced sensitivity of TPS spectroscopy to biomaterials

**Figure 7:**
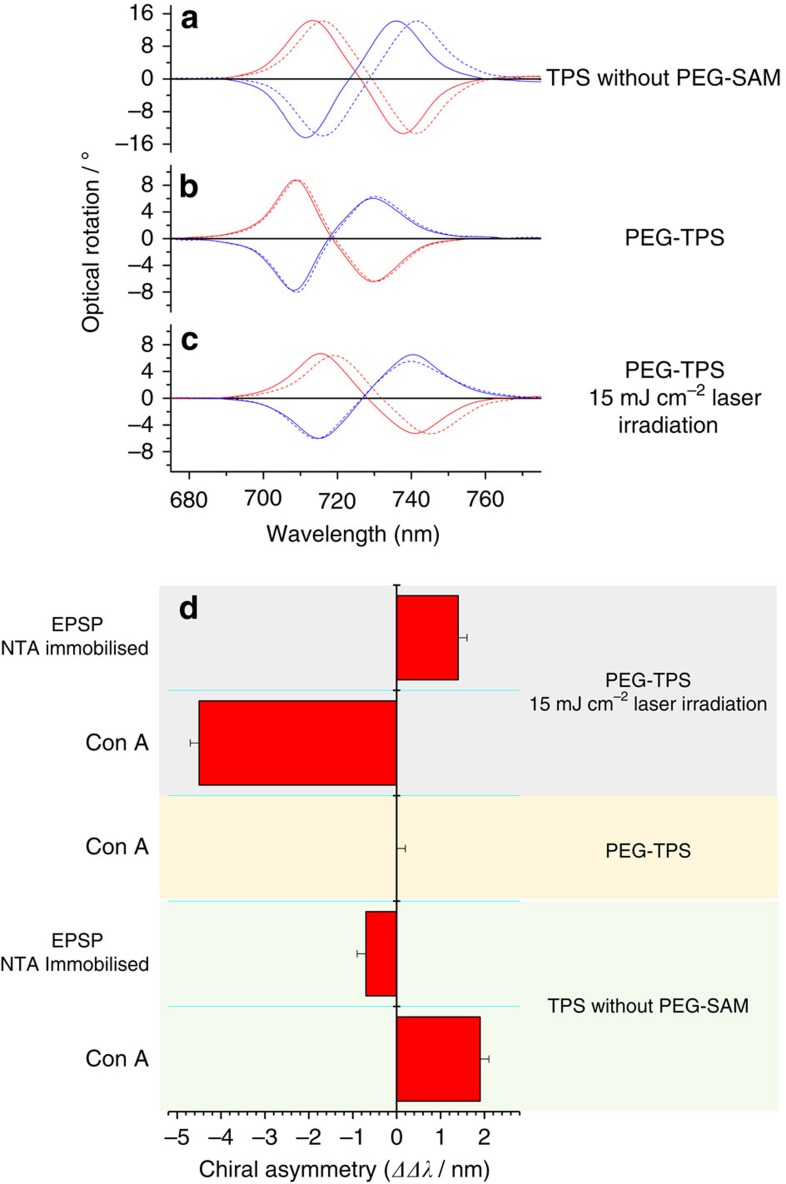
ORD shifts from buffer (solid) to ConA (dotted). (**a**) TPS, (**b**) PEG-TPS but not irradiated and (**c**) irradiated PEG-TPS. Blue lines represent right-handed structures and red represents left-handed structures. (**d**) *ΔΔλ* results for ConA and EPSPS protein experiments.

The elongated *trans*-PEG-SAM in the arms should allow adsorption of biomolecules, whereas the helical PEG-SAM will inhibit adsorption, essentially creating ‘nanopockets' in the arms. This ability of the PEG-SAM to selectively vary spatial chemistry provides a powerful tool for enhancing the functionality of nanomaterials. As an exemplar of the potential applications, we used the nanopockets created on the TPSs, to achieve detection of proteins at the attomole sensitivity. Specifically, the chiral evanescent fields that occupy the nanopockets were used to perform a form of polarimetry, plasmonic polarimetry^32^, which measures the asymmetry of the effective refractive indices of chiral media on the handedness of the applied chiral evanescent field^2^. The asymmetries in refractive indices of chiral media can be parameterized by *ΔΔλ*=*Δλ*_R_*−Δλ*_L_, where *Δλ*_R/L_ is the shift in the wavelengths of the ORD resonances induced by the adsorption of a chiral medium. The *ΔΔλ* parameter is analogous to the optical rotation measured with conventional optical polarimetry. We performed plasmonic polarimetry using bare PEG-TPSs and laser-irradiated PEG-TPSs. The ORD spectra collected for the left- and right-handed forms of the three different types of TPSs in buffer (reference value) and solution of a model protein, Concanavalin A (ConA), and the *ΔΔλ* values extracted from these spectra for the peak labelled are shown in Fig. 7d. ConA was chosen as an exemplar system, because previous work has shown that it displays a large plasmonic polarimetry response^2^.

As expected, given that the helical PEG structure inhibits the adsorption of biomaterials, the ConA cannot be detected using non-irradiated PEG-TPSs. In contrast, *ΔΔλ* values of 1.9 and −4.5 nm are observed for the bare and laser-irradiated substrates, respectively. The change in sign of the *ΔΔλ* values on going from bare to laser-irradiated PEG-TPSs can be correlated with the sign of the overall integrated optical chirality of areas available for protein adsorption on both substrates (Supplementary Fig. 2). In the case of the bare substrate, protein will be exposed to evanescent fields with an overall negative integrated optical chirality. In contrast, for the laser-irradiated PEG-TPS, ConA will only be adsorbed in the arms of the structure and thus will experience fields with an overall positive optical chirality. The *ΔΔλ* parameter decreases with decreasing laser fluence and hence smaller areas of thermally transformed PEG-SAM, as might be expected (see Supplementary Fig. 9).

In a second series of experiments, we functionalized the deprotected arms using a procedure first proposed by Sigal *et al*.^33^, which enables recombinant histidine-tagged (His-tagged) proteins to be selectively immobilized to SAMs featuring a nitrilotriacetic acid (NTA) group co-ordinated to Ni(II). In particular, we have co-self-assembled a thiol-functionalized NTA derivative (NTA-thiol) and triethylene glycol mono-11-mercaptoundecyl ether (EG-thiol) spacer unit to create binding sites for the His-tagged protein 5-enolpyruvylshikimate-3-phosphate (EPSPS) synthase (see Supplementary Fig. 10) within the nanopockets. For comparison, we functionalized a bare TPS with the NTA-thiol/EG-thiol and immobilized the EPSPS across the whole surface. The *ΔΔλ* values obtained for EPSPS immobilized across the whole of the TPS and just in the nanopockets were −0.7 and 1.4 nm, respectively. The behaviour of EPSPS is qualitatively the same as for ConA, with laser-processed substrates having *ΔΔλ* values that are larger and of the opposite sign to those obtained when protein covers the entire surface.

In our experiments, we measure a single array of our nanostructures, which is roughly a square with 300 μm sides. Assuming that the irreversible transformation of PEG takes place in the arms of all nanostructures, we estimate that the total deprotected area in a single array would be 6.65 × 10^3^ μm^2^ (calculations in Supplementary Note 7). We only detect surface-bound molecules and hence can consider the total number of particles bound on our deprotected areas as those being detected. In the case of EPSPS, the molecules are His-tagged and chemically bound to the surface with no proteins present in the buffer during measurements. We estimate that for a plain TPS, the EPSPS contributing to the measured *ΔΔλ* values would be 1.46 femtomoles, whereas only 108 attomoles (∼6.5 × 10^7^ protein molecules) of EPSPS protein are being detected using our protection/deprotection strategy (calculations in Supplementary Note 7). In the case of ConA, we estimate that only 80 amol (∼4.8 × 10^7^ protein molecules) contribute to the measured *ΔΔλ* values (see Supplementary Note 7). Hence, a functionalized PEG-TPS allows us to detect attomole (∼6.0 × 10^5^ molecules) quantities and with enhanced performance from plasmonic polarimetry.

## Discussion

In conclusion, we demonstrate a novel strategy to achieve spatially selective surface functionalization on a nanostructure. It is based on a protection/deprotection strategy, which is rapid and can chemically modify macroscopic areas of substrates. The spatially selective deprotection step is driven by the localized water heating-induced unravelling of an initially helical protective thiol in selective areas of a nanostructure. The unravelled regions are both less densely packed, thus can be subsequently functionalized with another thiol and do not inhibit biomolecule adsorption. The strategy has allowed us to enhance the sensitivity of a biosensor enabling analyte molecules to be selectively located in regions with EM fields of high net chiral asymmetry. Using thermal gradients in water (or any solvent) to control surface chemistry of nanoscale regions over macroscopic areas is a versatile route for the high-throughput production of advanced functional materials.

## Methods

### Fabrication of TPSs

The templated substrates were prepared by a combination of high-resolution electron beam lithography and injection moulding. In brief, clean silicon substrates were coated with 80 nm of PMMA (Elvacite 2041, Lucite International) and exposed in a Vistec VB6 UHR EWF lithography tool operating at 100 kV. After exposure, the substrates were developed and submitted for electroplating, where a 300-μm-thick nickel shim was formed^34^. The shim was trimmed and mounted in a custom-made tool capable of manufacturing ASA standard polymer slides. An Engel Victory Tech 28 tons injection moulding machine was used in fully automatic production mode in the manufacture of the polymer slides using polycarbonate (Makrolon DP2015) as feedstock. Polycarbonate is used as a substrate material, because it is known to have the best ability to replicate the nanofeatures and is commonly used in the industry for optical storage media^35^. This process enables us to make more than 200 substrates per hour. The injection-moulded substrates have the chiral nanostructures imparted in the plastic surface and are subsequently covered by a continuous 100-nm Au film to complete the TPS process.

### PEG-SAM functionalization and ConA solutions

PEG-thiol (Sigma-Aldrich) was used to make a 833-μM solution in 95% ethanol and the TPS was allowed to self-assemble onto the nanostructure for 18 h. After being removed from solution, the TPSs were washed with water and dried under nitrogen. ConA (Sigma-Aldrich) was prepared in solutions at a concentration of 1 mg ml^−1^ (37.7 μM) using a 10-mM Tris/HCl buffer at pH 7.4. Surface plasmon resonance measurements to determine surface protein coverage were done using a Biacore 2000 Instrument (GE Lifesciences).

### Preparation of the NTA-thiol and His-tagged EPSPS samples

NTA-thiol was purchased from Prochimia and EG-thiol was purchased from Sigma-Aldrich. The preparation for the NTA-thiol/EG-thiol monolayer was performed similar to that described by Sigal *et al*.^33^.

An existing His-tagged *Escherica coli aroA* gene cloned into a pET 22b vector was transformed and overexpressed in BL21 star cells. One litre of liquid Luria broth media was used to grow the cells and protein expression was induced with isopropyl β-D-1-thiogalactopyranoside as an optical density of 0.6 at 600 nm. The cells were harvested 4 h after induction by centrifugation and resuspended in 50 mM Tris/HCl buffer pH 7.5. The cells were lysed using sonication and centrifuged at 25 K to remove all insoluble matter. The protein was purified from the cell lysate by NTA nickel affinity chromatography with the EPSPS protein eluted using 300 mM imidazole, 0.5 M NaCl in 20 mM Tris/HCl buffer pH 7.5, giving a protein solution with a concentration of 434.8 μM. This resulted in a yield of over 50 mg of protein. The protein was concentrated by centrifugal concentration and buffer exchanged by dialysis to make a final solution of 20 mg ml^−1^ EPSPS in 50 mM Tris/HCl buffer pH 7.5. The purity of the protein was assessed by SDS–PAGE and the enzyme activity was assessed using a phosphate release assay^36^.

HEPES-buffered saline (HBS) used in the EPSPS experiments were 10 mM HEPES and 150 mM NaCl in water adjusted to pH 7.4.

The EPSPS solution was made using EPSP synthase in 50 mM Tris/HCl buffer of pH 7.5 with a concentration of 4 mg ml^−1^.

After SAMs were fabricated from NTA-thiol/EG-thiol, measurements were taken using HBS for buffer values. The EPSP solution was left for 2 h and then rinsed with HBS before measurements were taken.

### EM field simulations

Numerical simulations of EM fields and thermal heat transfer were performed using a commercial finite-element package (COMSOL v4.4, Wave optics module with Multiphysics and a heat transfer module). Permittivity values for gold were taken from Palik's optical constants^37^. Drude broadening was applied using the method described by Kuzyk *et al*.^38^. Earlier work by Bouillard *et al*.^39^ shows that the variation in dielectric properties over the temperatures associated to our simulation are insignificant (<0.2%) and have hence been neglected. Periodic boundary conditions were used to emulate the array of nanostructures. Linear, polarized EM wave was applied at normal incidence through the polycarbonate substrate onto the structure. A subsequent heat transfer module was then used with the total heat dissipation from the EM model used as the heat source. A time-dependant function was applied to the total heat dissipation to create a heat source that would replicate a temporally square-shaped laser pulse as the source for EM heating. We used the appropriate thermal conductivities, heat capacity and density for the dielectrics and the metals. For the values used and further information on the simulations, see Supplementary Method and Supplementary Table 1.

### ORD and laser irradiation

We have used a custom-made polarimeter that measures the reflected light from our samples. It uses a tungsten halogen light source (Thorlabs), Glan-Thompson polarizers (Thorlabs) and a × 10 objective (Olympus). The samples are positioned with the help of a camera (Thorlabs, DCC1645C) and the spectrum is measured using a compact spectrometer (Ocean optics USB4000). Using Stokes methods, we can measure the intensity of light at four angles of the analyser and calculate the optical rotation dispersion of our chiral plasmonic arrays.

A nanosecond (8 ns)-pulsed Nd:YAG laser (Spectra Physics Quanta Ray) operating at a 10-Hz repetition rate was used for the irradiation. The sample was irradiated at normal incidence S-polarized light, using an unfocussed beam with an area of 1 cm^2^, for 1 min. Fluence was varied where stated. For all protein experiments, the fluence used was 15 mJ cm^−2^.

## Additional information

**How to cite this article:** Jack, C. *et al*. Spatial control of chemical processes on nanostructures through nano-localized water heating. *Nat. Commun.* 7:10946 doi: 10.1038/ncomms10946 (2016).

## Supplementary Material

Supplementary Information Supplementary Figures 1-11, Supplementary Table 1, Supplementary Notes 1-7, Supplementary Methods and Supplementary References.
