# Supplementary material for: Spatial control of chemical processes on nanostructures through nano-localized water heating
Source: Nat Commun. 2016 Mar 10;7:10946. doi: 10.1038/ncomms10946 (PMC4792951; doi:10.1038/ncomms10946)
Supplement: Supplementary Information — Supplementary Figures 1-11, Supplementary Table 1, Supplementary Notes 1-7, Supplementary Methods and Supplementary References. [file ncomms10946-s1.pdf]

Supplementary Figures

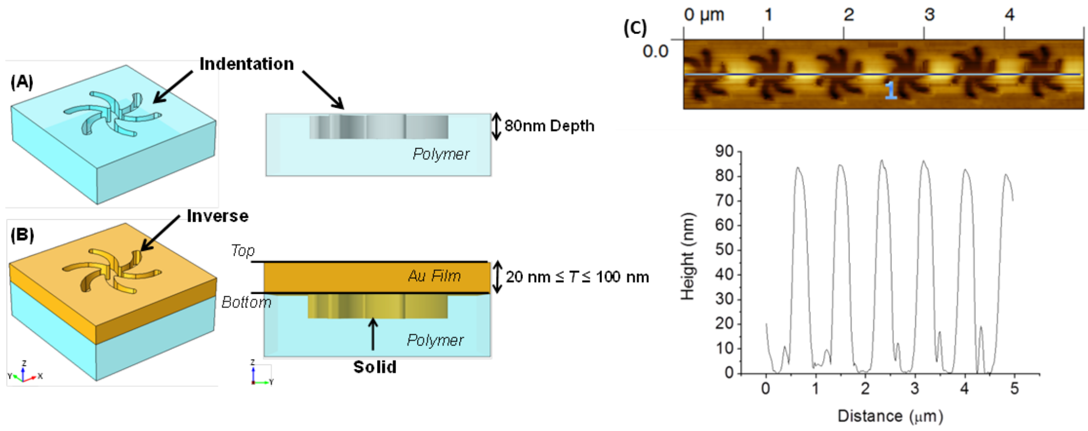

Supplementary Figure 1: **<u>Geometry of the nano indentation.</u>** Graphic of (A) Polymer substrate and (B) polymer substrate with Au evaporated to create a hybrid plasmonic structure. Atomic force microscopy results of a TPS without Au coating. The AFM image (C top) shows a left handed substrate. The plot (C lower) shows the measured height across the line marked 1 in the upper AFM image. The average height of the peaks in the plot is 84.18 nm

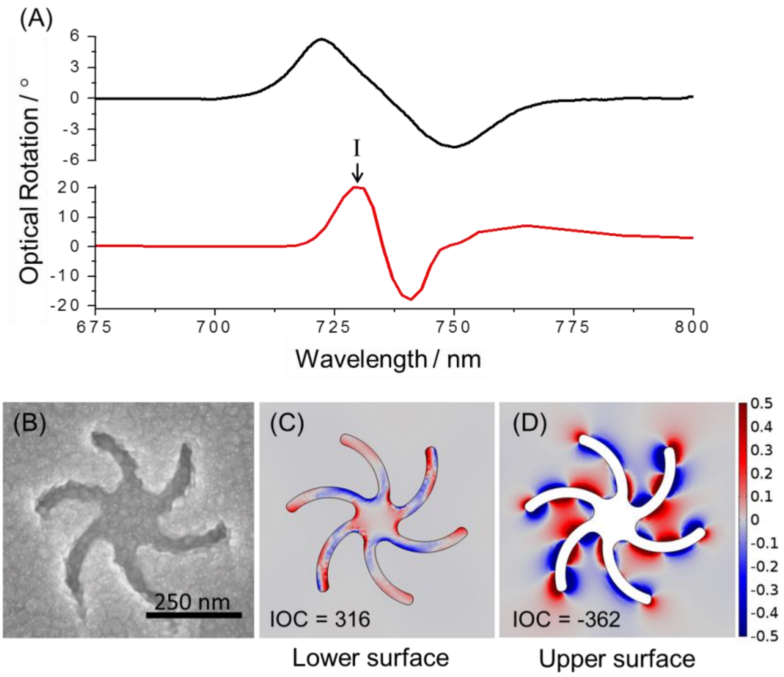

Supplementary Figure 2: **<u>Chiroptical properties of shuriken TPS</u>** (A) ORD experimental (black) and simulation (red). (B) SEM image of shuriken. Chirality surface plots for (C) lower and (D) upper surface.

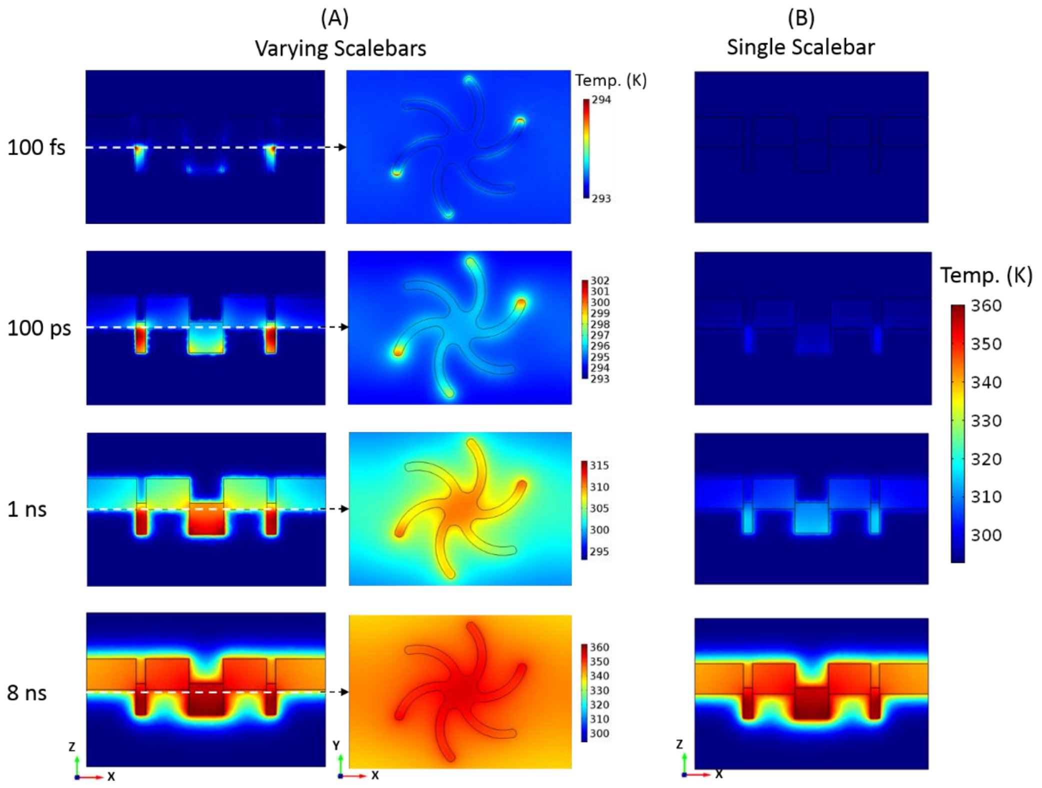

Supplementary Figure 3: **<u>TPS thermal gradient simulations.</u>** Thermal gradients in metal nanostructure with 8 ns laser pulse excitation. (A) Scale bars adjusted to show localisation (B) with scale bar range fixed for 8 ns.

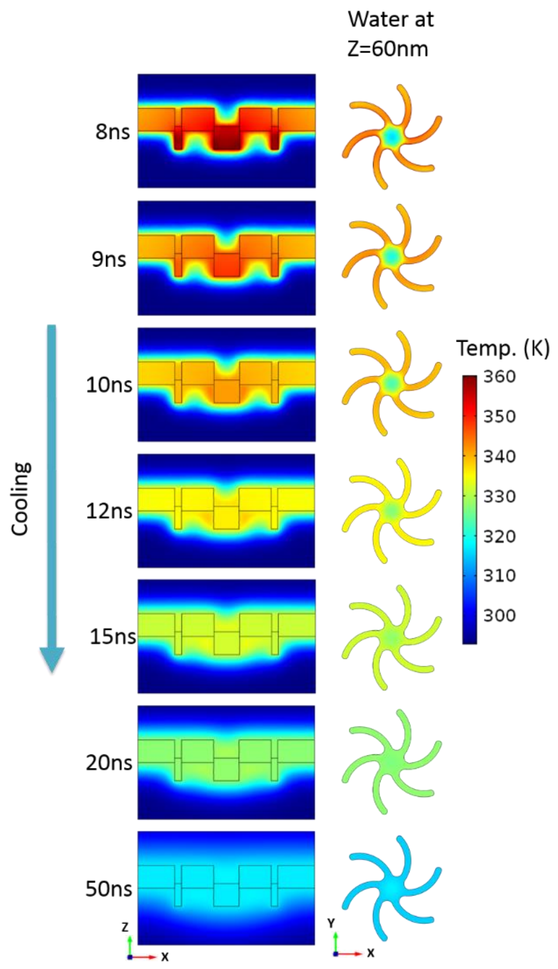

Supplementary Figure 4: **Post irradiation thermal simulations.** Temperature plots showing behaviour after the laser pulse has ended.

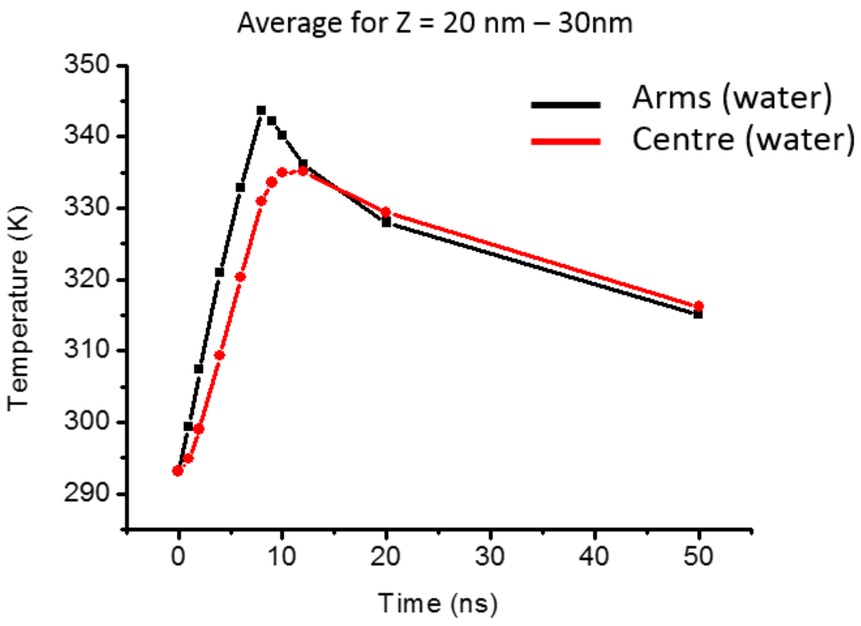

Supplementary Figure 5: **Regional thermal behaviour.** Average temperature of water in arms and centre between Z =20 nm and 30 nm.

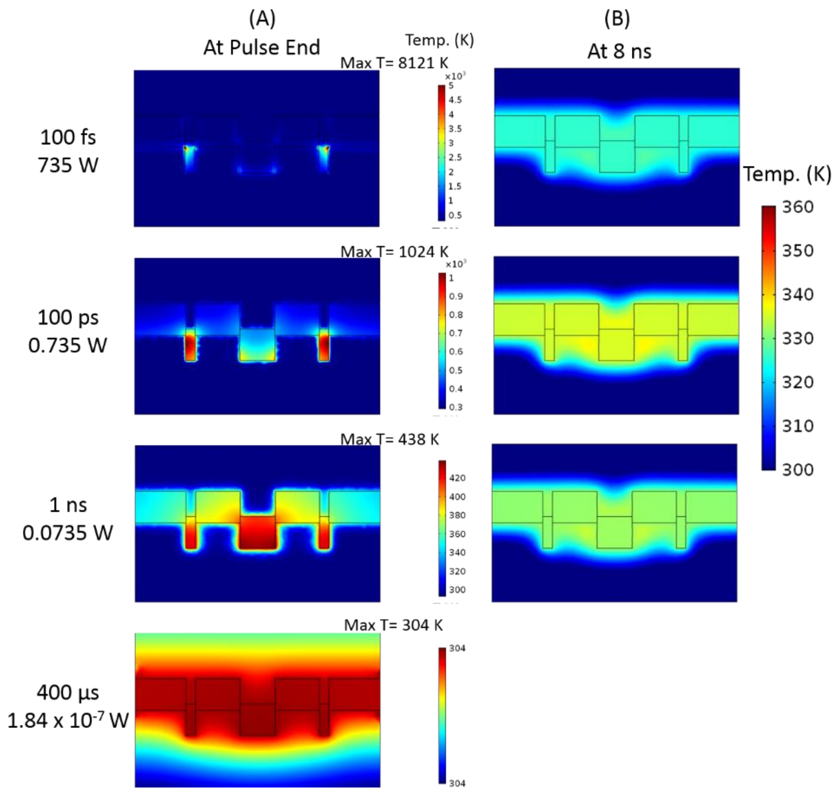

Supplementary Figure 6: <u>Pulse duration and power simulations.</u> Temperature plots of simulations using varying laser pulse durations and peak power (A) at pulse end, (B) at 8 ns.

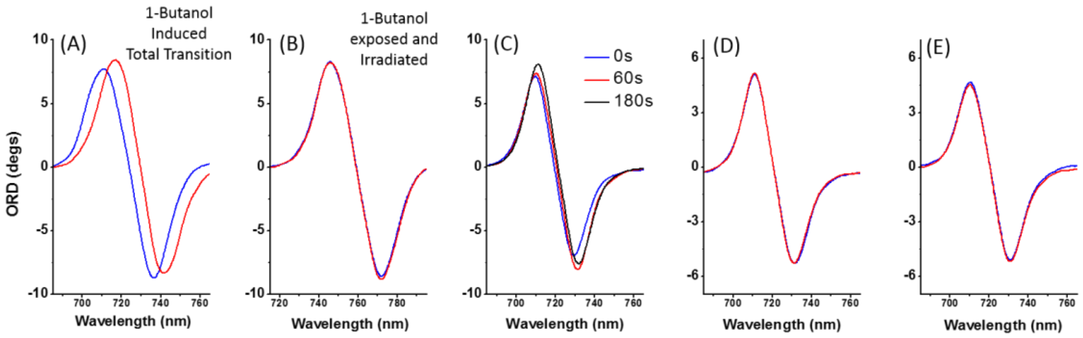

Supplementary Figure 7: <u>PEG-TPS ORD results.</u> (A) total transition due to exposure to 1-butanol; (B) further irradiation (in water) of PEG-TPS that has been exposed to 1-butanol, measured in 1-butanol; (C) varying exposure times for 8ns laser at 15 mJcm^{-2} fluence; and (D) irradiating the PEG-TPS in air using a 8ns laser at 15 mJcm^{-2} fluence. (E) PEG-TPS exposed to water at 323 K for 30 mins. Blue lines show ORD at start and red lines show ORD after the experiment.

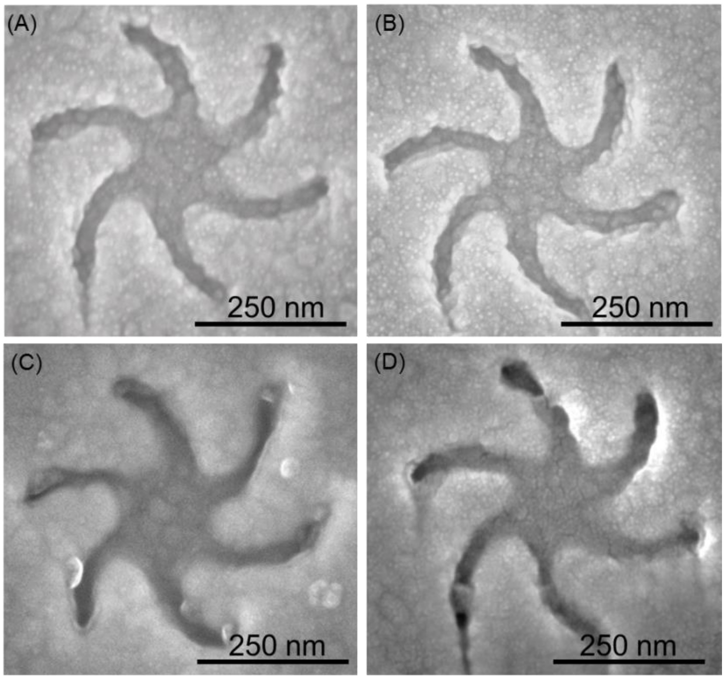

Supplementary Figure 8: **PEG-TPS SEM comparison.** SEM image of Au coated TPS without PEG-thiol that has (A) not been irradiated and (B) has been irradiated, showing no visible deformation. (C) PEG-TPS without irradiation and (D) PEG-TPS post irradiation.

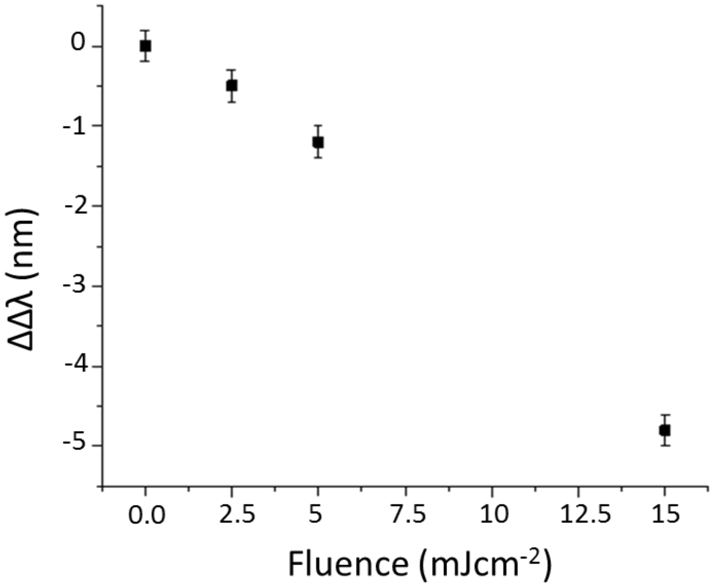

Supplementary Figure 9: Protein asymmetry fluence dependency. A plot of the $\Delta\Delta\lambda$ chiral asymmetry parameter observed for Con A as a function of laser fluence.

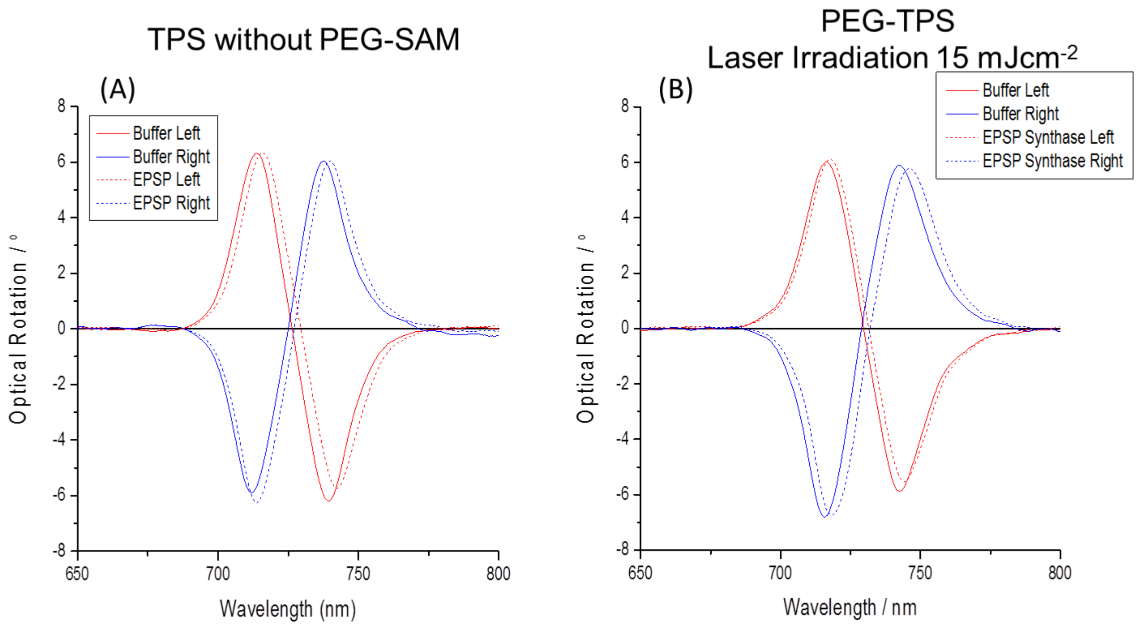

Supplementary Figure 10: **Experimental EPSP ORD data.** EPSPS experiment using (A) TPS without the PEG-SAM and (B) using a PEG-TPS.

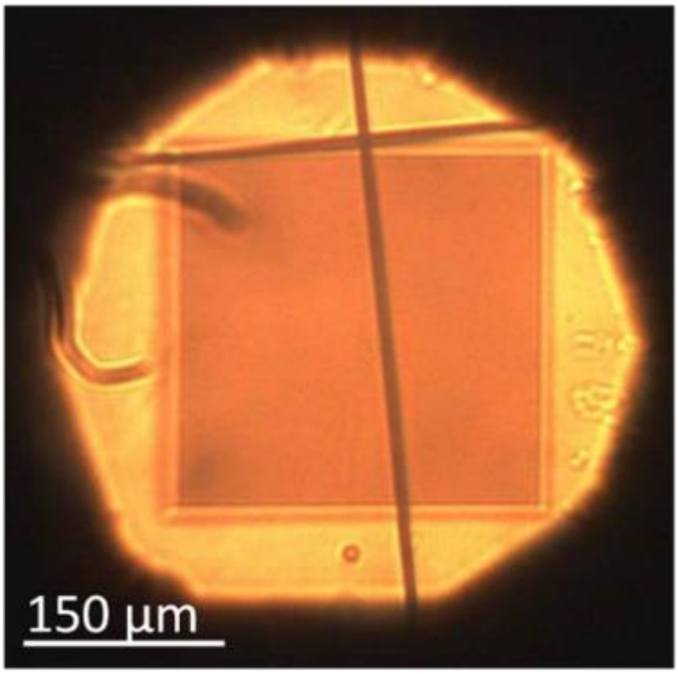

Supplementary Figure 11: **Optical microscope TPS image.** A single nanostructure array as viewed under our custom microscope. The array region is a 300 µm sided square.

Supplementary Tables

	$k\ (W\ m^{-1}K^{-1})$	$\rho\ (kg\ m^{-3})$	$C_p\ (J\ kg^{-1}K^{-1})$
Gold	318	19300	129
Water	0.6	999.9	4181.3
Polycarbonate	0.2	1210	1300

Supplementary Table 1: Heat related parameters for gold, water, and polycarbonate utilised by our simulation.

Supplementary Notes

Supplementary Note 1: Simulation results for Chirality

The chirality of the evanescent fields of frequency ω have been previously quantified using the *optical chirality* parameter *C*, Equation S1, first proposed by Tang *et al* [1]*:*

$$C = -\frac{\epsilon_0}{2}\omega\ Im(\boldsymbol{E}^* \cdot \boldsymbol{B}) \quad (1)$$

Where ϵ_0 is the permittivity of free space, ω is the angular frequency, $\boldsymbol{E}^*$ is the conjugate of the complex electric field vector, and $\boldsymbol{B}$ is the complex magnetic field vector. We have simulated the shuriken structure with normal incident light from the front end (similar to our experimental ORD measurements), Supplementary Figure 2 (A). The chirality surface plots shown in Supplementary Figure 2 (C) and (D), show that the chirality varies spatially. The integrated optical chirality (IOC) is simply the sum of all the chirality values shown in the plot over that surface. By spatially binding molecules in certain regions, we can expose it to certain fields more than others.

Supplementary Note 2: Thermal Gradients

Our calculations on the spatially-resolved time dynamics in our structures provide useful insight into the thermal diffusion in an optically-excited plasmonic nanostructure. As expected, the temperature gradients in the metal are small for 8 ns laser pulse excitation. This can be explained from the simple estimate of the thermal diffusion time inside a gold nanostructured film: The time for thermal diffusion via the gold from the centre to the end of the arm is estimated as $\cong L^2/D_t$, where the distance L=250 nm and thermal diffusivity of gold D_t= 1.27 x 10^{-4} m^2 s^{-1} and we get $t \approx 0.5$ ns that is much shorter than the pulse duration of 8 ns in our experiment. Therefore, we expect that temperature gradients inside the gold will be relatively weak. Supplementary Figure 3 shows a

temporal plot of the temperatures within the nanostructure for our 8 ns laser pulse. While in the femtosecond regime, highly localized temperature increase does occur, though the temperature gradients in the structure are extremely small. By 1 ns the lower structure has achieved thermal equilibrium (though temperatures still below 320 K) and by 8 ns the entire structure has temperatures inside ~350 -360 K range.

After the laser pulse ends, we consider the cooling behavior of the structure and the water around it, Supplementary Figure 4. While the metal nanostructure has achieved a near thermal equilibrium after 8 ns, the surrounding water still has significant (>20 K) thermal gradients that are decreasing with time. Around 15 ns, the temperature differences in the water and the metal have become negligible and the surrounding water and metal reach thermal homogeneity. The bulk water however remains significantly cooler and further cools the region.

Supplementary Figure 5 shows the average temperature in the water between Z = 20 nm and 30 nm for the arms and the centre. The gradient in the water is still substantial and the arms have significantly higher temperatures for longer, even when close to the metal bottom surface that will cause substantial elongation and intertwining of the PEG molecules in the arm regions.

Simulations for various laser pulse durations with varying peak power (but for the same fluence) were performed to show how thermal gradient formation is dependent on the pulse duration, supplementary Figure 6. When we consider the thermal plot at the pulse end time, the shorter pulses show the largest thermal gradients with very large maximum temperatures localised in the metal arms. Measurements at 8 ns are used as a reference and at this point the temperatures have equilibrated over the structure in all cases.

Supplementary Note 3: T TPS and PEG-TPS Characterisation Pre and Post Irradiation.

The TPS does not undergo any deformations before or after irradiation, Supplementary Figure 7, with or without the PEG-SAM on the surface. Therefore we conclude that we can negate any possible changes in the optical behaviour due to geometrical deformations.

Supplementary Note 4: PEG-TPS Transition data

Following the discussion on PEG-SAM transition from helical to trans forms, Supplementary Figure 8 shows further experiments to support the partial transition when laser irradiated. Exposure to 1-butanol will cause a full transition of all the PEG-SAM on the surface of the PEG-TPS, Supplementary Figure 8 (A). When a complete transitioned PEG-TPS (using 1-butanol) is further irradiated (8ns laser at 15 $mJcm^{-2}$ fluence) with water as the medium, we see no change, Supplementary Figure 8 (B). When a PEG-TPS is irradiated for several seconds, we find that the change (peak near 730nm) reaches saturation, and further heating has no significant effect, Supplementary Figure 8 (C). Lastly, heating a PEG-TPS in air only causes no change indicating that the process is water mediated, Supplementary Figure 8(D).

Supplementary Note 5: Effect of Fluence on $\Delta\Delta\lambda$ for Concanavalin A

The effect on the magnitude of the Concanavalin A chiral asymmetry parameter $\Delta\Delta\lambda$ is detailed in supplementary figure 9. This value of this asymmetry parameter is found to increase with increasing laser fluence. This is to be expected as a greater fluence results in larger regions of thermally transformed PEG-thiol hence more of the nanostructure surface is available for protein binding.

Supplementary Note 6: EPSPS Experimental Results

Supplementary Figure 10 shows results for the EPSPS binding experiment, comparing results using a TPS without PEG-SAM and using a PEG-TPS. The EPSPS results display the same qualitative behaviour as the Concanavalin A data, that is, the magnitude of the $\Delta\Delta\lambda$ parameter is larger for the PEG-TPS and is also of the opposite sign compared with clean TPSs.

Supplementary Note 7: Protein Immobilization and Estimation of Protein Mass on Measured Surface

Supplementary Figure 11 shows the array that we use for our measurements which has an area of approximately $90x10^3$ μm^2. If we consider that ideally an entire arm will be functionalised, and given we have arms with an approximate length of 150 nm and 40 nm wide, each arm has an area of 0.006 μm^2. For six arms this would be a total area of 0.036 μm^2. Our array has 430 by 430 shurikens in the vertical and horizontal directions giving us a total of 184900 shurikens per array. Hence the total area, functionalised in a single array such as that shown in Supplementary Figure 11 is approximately $6.65x10^3$ μm^2, reducing the total area contributing to our measurement by a factor of ~13.5.

The EPSPS used was a recombinant histidine tagged (His-tagged) protein that was purified using the His-tag by Ni^{2+} affinity and have good binding characteristics for the Ni^{2+} coordinated by a nitrilotriacetic acid (NTA) moiety. We have co-self-assembled a thiol-functionalized NTA derivative (NTA-thiol) and triethylene glycol mono-11-mercaptoundecyl ether (EG-thiol) spacer unit to create separate binding sites for the his-tagged protein, enabling them to be immobilized on a self-assembled monolayer (SAM) on the Au surface of the TPS.

Each EPSPS molecule will have approximately a minimum coverage area of 0.0001 μm^2 (10 x 10 nm), based on information available in the protein data bank. Hence 6.65×10^{7} protein molecules will occupy the functionalised pockets and contribute to our measurements using an irradiated/deprotected PEG-TPS array. EPSPS has a mass of 46 kDa or 7.6×10^{-20} g, hence the total mass of the surface bound protein would be ~ ~5 pg or 108 attomoles. For a plain TPS, the total bound protein would be ~1.46 attomoles. Given that the concentration of NTA to the spacer molecule is 1:9, the total mass of our surface bound protein will be even less, in this case roughly 12 attomoles in contrast to 162 attomoles for a plain TPS.

In the case for Con A, using surface plasmon resonance (SPR) measurements (Biacore 2000) we have determined that 1281×10^{-6} pgμm^{-2} of Con A adsorbs on a bare Au substrate. Hence, assuming the same amount adsorb per unit area, for pockets which have a total area of only 6.65×10^{3} μm^2, we have ~8.5 pg of Con A adsorbed where the fields can interact and detect it. Con A has a mass of 106 KDa (a homotetramer) and hence we detect ~80 attomoles of Con A using a deprotected PEG-TPS.

Supplementary Methods

Electromagnetic Simulations

We have performed our numerical simulation of Maxwell's equations by using finite element approach with COMSOL v4.4 Multiphysics software, Wave optics module and a Heat transfer module. Similar to the experiment, S-polarised light is incident normal to the structure through the polycarbonate substrate for laser heating simulations, and from the front for the ORD and chirality results. The heat transfer module used the total heat dissipation from the EM model (transferred using Multiphysics Electromagnetic heat source) as the heat source. A time dependant function was applied to the total heat dissipation to create a heat source that would replicate a square shaped laser pulse as the source for EM heating. The ORD has been calculated using the far field electric field components and applying Stokes method. Periodic boundary conditions were used on all four side boundaries to account for the periodicity of the nanostructures with perfectly matched layers

behind the input and output ports, (top and bottom). We used the same approach as in Kuzyk, A. *et al.* [2], to apply Drude model accompanied by an additional inhomogeneous broadening parameter for modification of bulk permittivity of Au. We used Palik's data [3] for experimental bulk permittivity of Au. The refractive index of water were taken from Hale et al [4]. The values for refractive index of polycarbonate were taken from Sultanova et al [5]. Earlier work by Bouillard et al [6], shows that the variation in dielectric properties over the temperatures associated to our simulation are insignificant (< 0.2%) and have hence been neglected. We used swept mesh for the perfectly matched layers and tetrahedral meshing for the nanostructures and its surrounding media with automated meshing limited to a maximum element size of 18nm in the metal and 30 nm in the surrounding media. We have tested this model in earlier work [7] and shown results for the optical properties (Supplementary Fig. 2). Having Maxwell's equations solved, the time dependent partial differential heat equation can be solved to determine local electrical heating in the entire system. The heat related parameters such as thermal conductivity (k), density (ρ), and heat capacity at constant pressure (C_p), used in our simulation, are listed in Supplementary Table 1. Finally, the local heat and temperature distribution were derived for the various time points for different values of laser fluence analogous to the experiment.

Polarimetry

The optical rotation measurements were done using a custom made polarimetry setup that can measure reflected light normal to the surface of the substrate. Linearly Polarised light is incident normal to the sample surface and the reflected light passes through an analyser. The intensity was measured using an Ocean Optics USB spectrophotometer. The analyser is rotated to measure the reflected spectra at four different angles; 0, 45, 90 and 135 degrees. These are then used to calculate the optical rotation (Ø) using the relation:

$$\emptyset(\lambda) = \frac{1}{2}\tan^{-1}\left(\frac{I_{45}-I_{135}}{I_0-I_{90}}\right) \qquad (2)$$

Where I is the intensity at the wavelength λ, and the subscripts 0, 45, 90 and 135 denote the analyser angle for the intensity measured. We can then obtain the optical rotation for each wavelength that provides the ORD.

Fabrication of Templated Plasmonic Substrates

The TPSs were made using and an injection moulding machine (ENGEL), following the technique explained by Gadegaard et al [8]. The master shim for this was made using electron beam lithography fabrication. For the master, 100 nm of PMMA was spin coated onto a Si wafer and baked for an hour at 180 ˚C. The resist was patterned using a VB6 UHR EWF lithography tool (Vistec) with the required pattern. The resist was then developed in IPA and Methyl Isobutyl Ketone, MIBK (3:1 ratio) for 60 secs. Ni was electroplated onto the surface and removed from the wafer to provide the Nickel shim that is then used as the master in a tool placed in the injection moulder. Polycarbonate pellets are then thermally heated and pushed into the tool to create small plastic slides with the nanostructures indented on the surface. These slides are then placed in an electron beam metal evaporator (Plassys MEB) where 100 nm of Au is coated onto the surface at a rate of ≈0.3 nm s^{-1}. This provides the TPSs which is then cleaned in an oxygen plasma asher for 30 sec before any experiments are performed.

The TPS are made by depositing gold on a nanopatterned surface. The nano patterns are actually indentations in the surface as shown in Supplementary Figure 1. The indentations have a depth of ~80 nm, are 500 nm in length from arm to arm, and have a pitch of 700 nm. When gold is evaporated onto the surface, it takes the shape of the indentation and forms a hybrid plasmonic structure constituting an inverse structure at the top and a solid one at the bottom. For more information on the plasmonic behaviour of the TPS refer to Karimullah et al [7].

Supplementary References

1. Tang, Y. & Cohen, A. E. Optical chirality and its interaction with matter. *Phys. Rev. Lett.* **104,** 163901 (2010).
2. Kuzyk, A. *et al.* Reconfigurable 3D plasmonic metamolecules. *Nat. Mater.* **13,** 1–5 (2014).
3. Palik, E. D. *Handbook of Optical Constants of Solids*. (Elsevier Science, 1998).
4. Hale, G. M. & Querry, M. R. Optical Constants of Water in the 200-nm to 200-μm Wavelength Region. *Appl. Opt.* **12,** 555 (1973).
5. Sultanova, N., Kasarova, S. & Nikolov, I. Dispersion properties of optical polymers. *Acta Phys. Pol. A* **116,** 585–587 (2009).
6. Bouillard, J. S. G., Dickson, W., O'Connor, D. P., Wurtz, G. a. & Zayats, A. V. Low-temperature plasmonics of metallic nanostructures. *Nano Lett.* **12,** 1561–1565 (2012).
7. Karimullah, A. S. *et al.* Disposable Plasmonics: Plastic Templated Plasmonic Metamaterials with Tunable Chirality. *Adv. Mater.* (2015). doi:10.1002/adma.201501816
8. Gadegaard, N., Mosler, S. & Larsen, N. B. Biomimetic polymer nanostructures by injection molding. *Macromol. Mater. Eng.* **288,** 76–83 (2003).
